# Improving nocturnal event monitoring in people with intellectual disability in community using an artificial intelligence camera

**DOI:** 10.1016/j.ebr.2023.100603

**Published:** 2023-04-23

**Authors:** Sarah Lennard, Rachel Newman, Brendan McLean, Caryn Jory, David Cox, Charlotte Young, Eve Corson, Rohit Shankar

**Affiliations:** a Department of Intellectual Disability Neuropsychiatry, Research Team, Cornwall Partnership NHS Foundation Trust, Truro TR1 3QB, UK; b Plymouth University, Peninsula School of Medicine and Dentistry, UK; c Cornwall Intellectual Disability Equitable Research (CIDER), University of Plymouth Peninsula School of Medicine, Truro TR1 3FF, UK

**Keywords:** Artificial intelligence, Developmental disabilities, SUDEP, Risk mitigation, Seizure deduction technology

## Abstract

There is higher prevalence of epilepsy and SUDEP in people with intellectual disability (PwID) compared to general population. Accurate seizure recording particularly at night can be challenging in PwID. Neuro Event Labs seizure monitoring (Nelli) uses high-quality video based artificial intelligence to detect and record possible nocturnal seizures. This study looks to evaluate the clinical utility and acceptability of Nelli in PwID and epilepsy. Family/carers of PwID and drug resistant epilepsy with suspicions of nocturnal seizures who had not tolerated routine or ambulatory EEGs were invited to evaluate Nelli. Relevant demographics and clinical characteristics were collected. Nelli’s impact, it’s facilitators, barriers and feedback quality was captured from patient and professional stakeholders. Quantitative and thematic analysis was undertaken. Fifteen PwID and epilepsy and four health professionals were involved. Nelli recorded 707 possible seizure events across the study cohort of which 247 were not heard or recognised by carers. Carers recorded 165 episodes of ‘restless’ or “seizure behaviour” which the Nelli did not deem to be seizures. There was 93% acceptability. Thematic analysis revealed three broad themes of device acceptability, result implementation and possible seizure recognition ability. Nelli allowed for improved communication and care planning in a hitherto difficult to investigate population.

## Highlights

• People with intellectual disability (PwID) have high prevalence of epilepsy & SUDEP.
• Accurate seizure recording particularly at night can be challenging in PwID.
• Neuro Event Labs (Nelli) video artificial intelligence possibly detects seizures.
• We evaluated Nelli’s Clinical utility in PwID (n = 15) in whom EEGs was not possible.
• Nelli allowed for improved care planning in a difficult to investigate population.

## 1 Introduction

Epilepsy is co-morbid in a quarter of people with intellectual disability (PwID) [1]. PwID with epilepsy are recognised to have worse health, social and quality of life outcomes including premature mortality compared to their peers without ID. Epilepsy in PwID is associated with a significant cognitive deficit, multimorbidity and polypharmacy [2]. Up to 70% of this population are considered treatment resistant and have seizures all their life [2].

Nocturnal seizures are an independent risk factor in sudden unexpected death in epilepsy (SUDEP), accounting for up to 70% of all SUDEPs [2], [3]. PwID and epilepsy are considered three times higher risk of SUDEP [3]. Night surveillance could contribute to preventing SUDEP, [4], [5].

Many PwID, due to their cognitive and communication deficits struggle to outline any nocturnal events. Clinicians are heavily reliant on family or carers to furnish information, which can be of differing quality [6]. Waking night care staff and family members may give some description of nocturnal activity but are often unable to accurately describe them [7]. Seizure diaries too are unreliable [7].

Automated technology in identifying seizures, by recognising visual cues from subtle behaviors such as facial, limb movements, repetitive movements, and sound, is developing [8], [9]. It has proved useful in in identifying events with differing semiology [10].

Gold standard diagnosis of epilepsy includes a suitable EEG and video telemetry [11]. However, for many PwID especially those with moderate to profound ID accessing EEGs particularly as a longitudinal investigation is practically impossible due to resource and distress issues. There is a need to explore other methods to monitor potential seizure activity, specifically at night, to validate diagnoses, understand frequency and intensity, mitigate risk, and improve quality of life [6].

The personal recording unit (Nelli) which comprises of a computer, a camera and a microphone produced by Neuro Events laboratory, (Tampere, Finland), is currently a prescription-only device indicated as an adjunct to seizure monitoring in a home or healthcare facility when the person with epilepsy sleeps [12]. The portable audio and video recording unit sends data either in real time or can be stored onto a data stick to transfer to a remote server. The system automatically analyzes the data to indicate motor events and categorise them as likely to be “epileptic” or “non-epileptic” in origin. The video recording is then sampled (8–9% of the recording) by a clinician with expertise in epilepsy, who by reviewing the semiology confirms the sensitivity and specificity of the event labelling. The system has been validated in a blinded setting against video-EEG monitoring at an epilepsy centre in Europe. It provided accurate identification of tonic-clonic, tonic and focal motor seizures, and is approved in Finland for clinical use by the government body [13], [14]. In a recent validation study, the Nelli® hybrid system was used in a blinded setting without any prior information of the patients or their seizure types, against video-EEG monitoring at another epilepsy centre in Europe. It provided accurate classification of major motor seizures including tonic–clonic, clonic, and focal motor seizures [15].

### 1.1 Aims

1. To evaluate the acceptability of Nelli being placed in the rooms of PwID, from the participant and carer perspective & understand the barriers and enablers to implementation from the healthcare professional perspective
2. To clarify the consistency of Nelli to identify episodes to help increase clinical suspicion of possible seizures

## 2 Methods

The evaluation took place in Cornwall (pop: 565,000,) a rural county of the United Kingdom, with an Epilepsy service having approximately 200 PwID with pharmacoresistant epilepsy. PwID who had high levels of suspicious seizure type events at night over the autumn / winter of 2020–2021 but not had their clinical epilepsy diagnoses supported by EEG, due to not tolerating routine or ambulatory EEGs were identified. Their family members or carers were approached for consent and to offer Nelli as a service evaluation.

For each participant, relevant demographics and clinical characteristics were collected. Supplementary information one shows what Nelli looks like. The researcher or nurse specialist conducted the setting up in each case. Neuro Event Labs team were available to check for positioning and recording. Training was given to the participants, care team or family member in the use of the equipment. The Neuro Event Labs team had no active role in patient selection and were blinded to the patient details and had no part in the analysis of the collected data.

The findings for each participant were compiled in a secure report (containing both video and semiological descriptions of events) and sent to the relevant health care professionals following completion of the study (See supplementary information two for an example).

A questionnaire was developed by the clinical team in discussion with experts by experience identifying preemptively what the key areas of inquiry need to be on the impact of Nelli. This was divided into two parts. Part A asked about the set-up and practicalities of the Nelli in the home environment specifically “set up and placement of Nelli”, “experience of Nelli”, and “satisfaction of results produced”. This was in the form of three questions in Likert style with a range of 7 options from “worst to best” (worst, very bad, bad, neither bad or good, good, very good, best). A further “yes or No” question i.e., “did the results give peace of mind” was also asked. Part B asked about how they feel about the Nelli feedback after they had had a consultation with the epilepsy specialist team using it’s results. The questionnaire administration was expected to take approximately 30 min per participant to complete.

### 2.1 Governance and Ethics

The project used anonymised pooled data from a single centre collected as part of a service evaluation/improvement and registered as such with the host NHS organisation. The NHS Health research authority tool confirmed no formal NHS Ethics approval was required [16]. Informed consent from participants was sought and where they lacked mental capacity to provide informed decision, assent was collected from a family member (parents/siblings). In addition, best interest assessments for the use of Nelli as outlined by the Mental Capacity Act 2005 were completed by the nurse specialists [17]. Information sheets were provided and for each considered participant the process was discussed in detail. All authors are clinically contracted to the service and were the only people privy to individual medical records.

### 2.2 Analysis

While no analysis of the event counts was done, the average mean of the quantitative feedback is reported. For each interview, detailed notes were captured and written up by the interviewer. The notes from each interview were reviewed broadly based on the reflexive and iterative process of verbatim transcription and analysis to ensure that all key messages were captured. A coding framework for analysis was developed collaboratively following an initial review of the data. This was then refined throughout the coding and analytical process. Once coded, data was analysed thematically to draw out key themes and findings. Descriptive thematic analysis is often used in healthcare research that has a strong link to clinical practice. The identified thematic results consist of a descriptive summary of the data to present it in an accessible way for readers under the main headings.

## 3 Results

One participant was able to consent and fourteen required the best interest/assent process from their families. Those involved in the direct care of the study cohort i.e., carers (15), family members (4), and health care professionals (4) completed the questionnaire. Setting up Nelli took approximately 30 min. Table 1 shows each participant’s diagnoses, medications, and nocturnal care availability. Table 2 identifies the quantitative feedback received from family /carers for part A of the survey.

**Table 1:** Participant information showing participant diagnoses, medications, nocturnal care team availability and length of time Nelli was in place.

Participant	Diagnoses	**Medications**	VNS	Professional Night Care team (Non-registered care assistants)			Family	Time period Nelli in place (nights)	Suspected nocturnal detections frequency	Total number of detection highlighted by Nelli
				Waking	Sleeping	Same Room	Same Room			
1	Autism, Intellectual Disability, Epilepsy, Sleep Apnoea	levetiracetam (LEV) topiramate			Y	N	N/A	14	Weekly	18
2	Lennox-Gastaut Syndrome, Intellectual Disability, Cortical Migration Disorder,	lamotrigine (LTG) clobazam Lacosamide	Y	N	N	N	N	14	Unknown, suspected nightly	61
3	Intellectual Disability, Epilepsy, Congenital agenesis of corpus callosum, Cardiac pacemaker visual impairment	As required midazolam (MDZ PRN)	N	N/A	N/A	N/A	Y	14	Nightly	7
4	Intellectual Disability, Neuronal Migration Disorder, Epilepsy	Tiagabine Clobazam PRN MDZ PRN	N	Y	N (parents sometimes	N	N	14	Nightly	45
5	Intellectual Disability, Challenging behaviour, Birth trauma with anoxic encephalopathy in and assisted delivery, Right hemiparesis, Hemianopia, Epilepsy	clonazepam LTG carbamazepine MDZ PRN	N	Y	Y	N	N/A	4 days then removed, following discussion with care team. Participant became distressed by camera and posed a safety risk.	Weekly	1
6	Intellectual Disability, Ring 20 Chromosome syndrome mosaic, Epilepsy	Clobazam rufinamide phenytoin capsules MDZ PRN	Y	Y		Y		14	Nightly	418
7	Intellectual Disability and epilepsy	VPA LTG	N	Y		N		14	Unknown	4
8	Intellectual Disability, Epilepsy and Autism	LTG VPA.	N	Y		N		14	Unknown	0
9	tuberous sclerosis, Intellectual Disability and epilepsy	Everolimus LTG Topiramate rufinamide MDZ PRN	Y		Y	N		20	Nightly	112
10	Intellectual Disability and epilepsy	Pregabalin clonazepam MDZ PRN PRN **–** Diazepam		Y		N		14	Weekly	22
11	Intellectual Disability, Tuberous Sclerosis complex, Epilepsy	CBZ, VPA, **c**lonazepam 0.2 mg (0.5 ml) at night MDZ PRN	N		Y	N	N	14	Nightly	12
12	Treatment-resistant epilepsy, Intellectual Disability, Autism, Sleep disorder	Zonisamide, VPA	N		Y	N		14	Unknown, suspected weekly	1
13	MECP2 Duplication Syndrome, Intellectual Disability, Epilepsy	VPA, LEV	N	Y		N		14	Nightly	6
14	Down’s Syndrome, Intellectual Disability, Dementia – Alzheimer’s, Epilepsy	VPA	N	Y		N		14	Weekly	0
15	Intellectual Disability, Epilepsy	VPA	N				N	14	Nightly	0

**Table 2:** Results from the quantitative feedback for survey Part A: Nelli – Participant, family, and carer feedback (n = 14).

Q 1 to 3	Score (1 to 7 – 1 being worst and 7 being best)
Set up and placement of Nelli	6.5/7 average
Experience of Nelli	6.3/7 average
Satisfaction of results produced	6.4/7 average
Q4 Did the results give peace of mind? (Y/N)	13/14 positive responses

Nelli was well accepted by 14 of 15 PwID and remained in place for all except one for a minimum of 14 nights. The decision was made to remove Nelli for one person to prevent injury and equipment damage.

Nelli recorded 707 possible seizure events across the 14 PwID of which 247 were either not heard or not recognised by carers. One participant had 50% of the possible seizure events missed by night care staff. Carers recorded 165 episodes of ‘restless’ or “seizure behaviour” which the Nelli did not deem to be seizures.

Thematic analysis of the interviews revealed three broad themes and the finding are presented in Table 3. Example comments are provided in supplementary information three. Key thematic analysis findings included both patient representatives and professionals giving positive feedback on the ease of Nelli installation, flexibility in its placement and added value in identifying improved insights to episodes qualitatively and quantitatively. Results of Nelli were found to be useful to improve holistic care. Carers particularly reported reduction in their stress. There were no concerns of privacy intrusion.

**Table 3:** Thematic analysis.

Theme	Family/Carer/Patient	Professional
*Acceptability of placement and results*	User could place the equipment with remote instruction gave peace of mind to family and carers helpful in informing others (i.e., sharing information with respite care etc.) as to what happens at night, The intrusion of privacy was accepted by family and carers once they saw the equipment, the ability to view test videos, and the ability to easily pause the recordings for personal care. One family member wrote a story to introduce the camera to the participant prior to installation, the camera was referred to the participant as the name given in the story to promote acceptance (supplementary information four).	The reporting of the results within the scatter graph showed the clinicians possible trends in seizure activity as well as length of activity (supplementary information two) Nelli could be integrated into clinical practice well, with the ability to review types of possible seizures and frequency at night. gave clarity of possible seizures which previously had often relied upon care staff reporting, which can be misleading as to whether the person had a possible seizure or not. The epilepsy nurse specialists were able to update seizure descriptions for those who took part with additional knowledge gained from the videos. The Neuro Event labs team reported of some challenges to match the characterisation of seizure types by Nelli with the information given by the carers finding that symptoms list did not match what was found during the recordings
*Implementation of results*	allowed for a comparison with reported daytime seizures looking at similarities and differences able to understand the need for further medication after the review of night-time events. Identified need to increase night waking staff, changing beds due to its texture and the It helped support review of night-time protocols for the carers – with reviewing safety, first aid for seizures, position of monitors and rationale for having an audio/visual monitor.	Identified more possible seizure activity than expected or reported by carers Was helpful to show team members an individual’s possible seizure pattern were highlighted as a learning curve. Helped identify gaps in surveillance, safety issues (banging of head on metal side bar of bed) , need for seizure first aid training and recognise correct administration of midazolam. Promoted alternate considerations such as sleep apnea
*clarify the consistency of Nelli to identify episodes to help increase clinical suspicion of possible seizures*		There were mixed results and it was generally felt that it could be placed higher than current standard care of using carer driven descriptors but lower than video telemetry and ambulatory EEG. However, it is recognised that video telemetry and ambulatory EEGs are specific diagnostic time limited resource, which might or might not pick up an event in the time it has been used for i.e., 24–72 h, while Nelli can give much broader picture given its longer time span and in a home environment. Feedback given by the clinical team prompted the categorisation of the ‘unspecific’ seizure type to be subdivided in to two different types i.e., Unspecific (possibly epileptic) and Other/Noteworthy.

## 4 Discussion

The implementation of nocturnal surveillance in PwID and epilepsy depends on the choice of technology and the cognitive ability of each individual, together with the carer views [11]. Technology use needs to be based on individual risk and ethical considerations of personal intrusion [11]. However, the fact that SUDEP deaths and seizure related harm linked to nocturnal seizures are more likely to be prevalent in PwID is a paramount consideration [6].

Nelli benefitted those who were not able to tolerate more traditional methods of seizure investigation and analysis. Nelli added to the overall picture for each person with ID and epilepsy to best support them to ensure they are safe at night. Nelli prompted discussions about possible need for waking night support or installation of monitor, environmental conditions, and other seizure related matter. Clinicians adjusted treatment plans post two weeks of Nelli recording. This was influenced by recognition by care teams of possible seizures being missed. Only one participant had no change post Nelli administration in their seizure management strategy.

The system appears to pick up the pre-ictal and early stages of a seizure such as breathing pattern change which was often missed by carers. This better-informed clinician of nocturnal events not only to treat but also to train carers in the possible seizure patterns of everyone thus improving their care plans. Nelli also identified possible non-seizure related issues, such as movements and behaviours misinterpreted as seizures, and other potentially relevant medical problems such as sleep apnoea. There was improved clocking of event frequencies.

Care providers generally responded favourably to Nelli with high levels of satisfaction and belief of it giving them “peace of mind”. The fact that caregivers reported “satisfaction” and “peace of mind” with the system is likely based on their assumption that the technology accurately identified seizures. Without confirmation of that accuracy, to some extent this satisfaction might be misplaced. However, it could be argued that the satisfaction perceived is a response to multi-faceted issues. It could be that the knowledge that finding out hitherto unknown episodes of concern is a relief. Similarly, where the individual was in care responsibility, the knowledge that carers were undertaking the needed duties to their best of their abilities would have been comforting. Most importantly Nelli provided insights on what and where to further investigate.

### Study limitations

Although this study identifies a real need for accurate seizure detection technology that does not rely on scalp EEG electrodes for PwID, it does not provide evidence that the Nelli system fills that need. It however is a step further in aiding a clinical diagnosis and management strategy in an otherwise difficult to assess and treat population.

Nelli “married up” with previously observed descriptions of possible nocturnal seizures. It seemed to either agree or disagree with these descriptions. In some instances, Nelli could not help health professionals establish if the witnessed motor activity was seizures or non-seizure related (for example autistic automatisms/stereotypies). It would have been useful to have ongoing EEG to validate the Nelli findings. However, the population selected was those who had not tolerated an EEG. It needs to be highlighted that this study was not designed to validate the accuracy of the Nelli system, as this has already been established elsewhere but in a non-ID population.

There is a need to do a well powered control study that compares this technology in this patient population with standard video-telemetry to provide further confidence in the diagnostic abilities of Nelli but the intolerance of external EEG monitoring in this population may never allow such an analysis to take place. We may thus have no option but to extrapolate the diagnostic utility from the existing patient database. It may however be possible to explore the technology in PwID who may be able to tolerate concomitant EEG and provide a much-needed community solution to the problem of differentiating possible seizures from other non-seizure events thus enhancing long term care in such complex clinical populations. Similarly, a cost analysis study could highlight if Nelli could reduce care costs by enhancing long term care.

It is also worth considering Nelli in perspective with current emerging thought lines of value of stand-alone video reviews being more helpful than EEGs for seizure detection [18]. A recent study has shown that stand alone smart phone recorded videos was a helpful adjunct in aiding seizure diagnosis conclusively [19]. A systematic review of 17 studies inquiring into the benefits of home video recording outlined the multiple benefits of it [18]. Not only did it consistently aid better clinical decision making it reduced stress levels in families and cost benefits [20]. Further, novel AI techniques are emerging to evaluate videos with suspect seizure episodes [21]. This suggests that similar technologies to Nelli can emerge with the added inclusion of AI to the video capture facilities which are now reasonably universal. Such developments can hopefully revolutionize and bring solutions to the current predicament and challenge of diagnosis and management of seizures in a difficult to engage population such as some PwID and epilepsy.

## 5 Conclusion

PwID and epilepsy have significantly worse health outcomes, increased, premature mortality and susceptible to irrational polypharmacy compared to their peers without epilepsy or those from general population with epilepsy [22], [23], [24], [25], [26]. It is imperative that new technologies be evaluated in a safe and ethical manner for the benefit of this group to improve their holistic outcomes.

There was high acceptability of Nelli in a complex and vulnerable patient cohort who could not tolerate traditional forms of investigations including EEGs. While the difficulty to diagnose seizures in PwID is clear, unfortunately for the aim of diagnosis, there is no gold standard/index standard with which to confirm accuracy. It would have been useful to have ambulatory EEG in the studied cohort to use as a comparison but understandably this is a very challenging group to maintain electrode contact. Also, semiologies in PwID are wildly heterogeneous. This makes feature extraction and classification limited by other AI databases in PWE without ID. In this regard, Nelli could offer potential for assisting diagnosis and possibly better management for improved safety and better resource utilisation in a difficult to support population.

*Ethics Statement*

The project used anonymised pooled data from a single centre collected as part of a service evaluation/improvement and registered as such with the host NHS organisation. The NHS Health research authority tool confirmed no formal NHS Ethics approval was required. Informed consent from participants was sought and where they lacked mental capacity to provide informed decision, assent was collected from a family member (parents/siblings). In addition, best interest assessments for the use of Nelli as outlined by the Mental Capacity Act 2005 were completed by the nurse specialists. Information sheets were provided and for each considered participant the process was discussed in detail. All authors are clinically contracted to the service and were the only people privy to individual medical records.

## Funding

This project was funded by Neuroevents Lab via an investigator-initiated grant proposal Neuroevents Lab had no input to the design, analysis or presentation of the results of this study. The first author, SL received researcher salary support from the investigator-initiated grant provided by Neuroevents Labs. BM has received institutional and research support from LivaNova, UCB, Eisai, Bial and Jazz pharma outside the submitted work. RS has received institutional and research support from LivaNova, UCB, Eisai, Veriton Pharma, Bial, Averelle and Jazz pharma outside the submitted work.

## Declaration of Competing Interest

The authors declare that they have no known competing financial interests or personal relationships that could have appeared to influence the work reported in this paper.

## References

[1] Robertson J., Hatton C., Emerson E., Baines S. (2015 Jul). Prevalence of epilepsy among people with intellectual disabilities: A systematic review. Seizure.

[2] Watkins L, O’Dwyer M, Kerr M, Scheepers M, Courtenay K, Shankar R. Quality improvement in the management of people with epilepsy and intellectual disability: the development of clinical guidance. Expert Opinion on Pharmacotherapy [Internet]. 2020 Feb 1 [cited 2022 Dec 20];21(2):173–81. Available from: https://pubmed.ncbi.nlm.nih.gov/31790280/. 10.1080/14656566.2019.1695780 31790280

[3] Young C., Shankar R., Palmer J., Craig J., Hargreaves C., McLean B. (2015 Feb). Does intellectual disability increase sudden unexpected death in epilepsy (SUDEP) risk?. Seizure.

[4] Maguire M.J., Jackson C.F., Marson A.G., Nevitt S.J. (2020 Apr 2). Treatments for the prevention of Sudden Unexpected Death in Epilepsy (SUDEP). Cochrane Database Syst Rev.

[5] Shankar R., Jalihal V., Walker M., Laugharne R., McLean B., Carlyon E. (2014 May). A community study in Cornwall UK of sudden unexpected death in epilepsy (SUDEP) in a 9-year population sample. Seizure.

[6] Young C., Shankar R., Henley W., Rose A., Cheatle K., Sander J.W. (2018 Sep). SUDEP and seizure safety communication: Assessing if people hear and act. Epilepsy Behav.

[7] Akman C.I., Montenegro M.A., Jacob S., Eck K., Chiriboga C., Gilliam F. (2009 Sep). Seizure frequency in children with epilepsy: Factors influencing accuracy and parental awareness. Seizure.

[8] Ojanen P., Knight A., Hakala A., Bondarchik J., Noachtar S., Peltola J. (2021 Jan). An integrative method to quantitatively detect nocturnal motor seizures. Epilepsy Res.

[9] An S., Kang C., Lee H.W. (2020 Jun 30). Artificial Intelligence and Computational Approaches for Epilepsy. Journal of Epilepsy Research.

[10] Ahmedt-Aristizabal D., Fookes C., Denman S., Nguyen K., Sridharan S., Dionisio S. (2019 Feb). Aberrant epileptic seizure identification: A computer vision perspective. Seizure.

[11] NICE. Overview | Epilepsies: diagnosis and management | Guidance | NICE [Internet]. Nice.org.uk. NICE; 2012 [cited 2022 Nov 22]. Available from: https://www.nice.org.uk/Guidance/CG137.

[12] Neuro Event Labs - Seizures. Clearly [Internet]. Neuroeventlabs.com. Neuro Event Labs; 2020 [cited 2022 Nov 23]. Available from: https://www.neuroeventlabs.com.

[13] Basnyat P., Mäkinen J., Saarinen J.T., Peltola J. (2022 Aug). Clinical utility of a video/audio-based epilepsy monitoring system Nelli. Epilepsy Behav.

[14] Peltola J., Basnyat P., Armand Larsen S., Østerkjærhuus T., Vinding Merinder T., Terney D. (2022). Semiautomated classification of nocturnal seizures using video recordings. Epilepsia.

[15] Armand Larsen A., Terney D., Østerkjerhuus T., Merinder T.V., Annala K., Knight A. (2022). Automated detection of nocturnal motor seizures using an audio-video system. Brain Behavior.

[16] Do I need NHS Ethics approval? [Internet]. www.hra-decisiontools.org.uk. [cited 2022 Oct 23]. Available from: http://www.hra-decisiontools.org.uk.

[17] Legislation.gov.uk. Mental Capacity Act 2005 [Internet]. Legislation.gov.uk. 2005. Available from: https://www.legislation.gov.uk/ukpga/2005/9/contents.

[18] Benbadis S.R. (2023). The Best Seizure Diagnostic Tool Is Not a Medical Device: Why Stand-Alone Video Review Needs a Current Procedural Terminology Code. Neurol Clin Pract.

[19] Amin U, Primiani CT, MacIver S, Rivera-Cruz A, Frontera AT Jr, Benbadis SR. Value of smartphone videos for diagnosis of seizures: Everyone owns half an epilepsy monitoring unit. *Epilepsia*. 2021;62(9):e135-e139. doi:10.1111/epi.17001. 10.1111/epi.17001 34254664

[20] Ricci L., Boscarino M., Assenza G., Tombini M., Lanzone J., Di Lazzaro V. (2021). Clinical utility of home videos for diagnosing epileptic seizures: a systematic review and practical recommendations for optimal and safe recording. Neurol Sci off J Ital Neurol Soc Ital Soc Clin Neurophysiol.

[21] Karácsony T., Loesch-Biffar A.M., Vollmar C., Rémi J., Noachtar S., Cunha J.P.S. (2022). Novel 3D video action recognition deep learning approach for near real time epileptic seizure classification. Sci Rep.

[22] Sun JJ, Perera B, Henley W, et al. Epilepsy related multimorbidity, polypharmacy and risks in adults with intellectual disabilities: a national study [published correction appears in J Neurol. 2022 Mar 5;:]. J Neurol. 2022;269(5):2750-2760. doi:10.1007/s00415-021-10938-3. 10.1007/s00415-021-10938-3 35067759

[23] Sun J.J., Watkins L., Henley W. (2023). Mortality risk in adults with intellectual disabilities and epilepsy: an England and Wales case–control study. J Neurol.

[24] Branford D., Shankar R. (2022). Antidepressant prescribing for adult people with an intellectual disability living in England. Br J Psychiatry J Ment Sci.

[25] Branford D., Sun J.J., Shankar R. (2023). Antiseizure medications prescribing for behavioural and psychiatric concerns in adults with an intellectual disability living in England. Br J Psychiatry.

[26] Branford D., Sun J.J., Burrows L., Shankar R. (2023). Patterns of anti-seizure medications prescribing in people with intellectual disability and epilepsy: a narrative review and analysis. Br J Clin Pharmacol.

