# Supplementary material for: Improving nocturnal event monitoring in people with intellectual disability in community using an artificial intelligence camera
Source: Epilepsy Behav Rep. 2023 Apr 23;22:100603. doi: 10.1016/j.ebr.2023.100603 (PMC10160340; doi:10.1016/j.ebr.2023.100603)
Supplement: Supplementary data 1 [file mmc1.docx]

**Figure 1 – Nelli: What does it look like?**


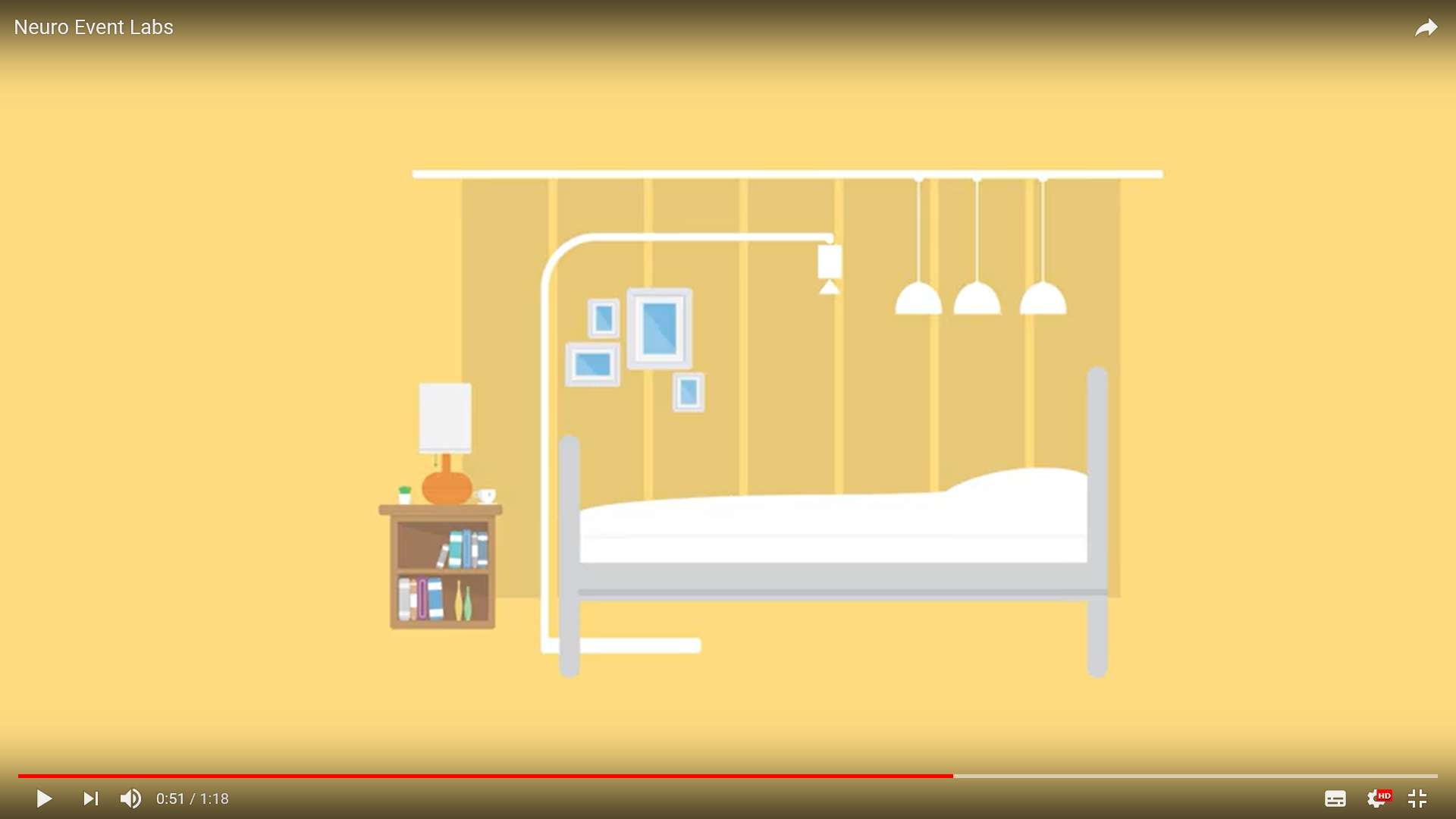


[**https://neuroeventlabs.com/for-patients**](https://neuroeventlabs.com/for-patients)

The equipment contains video camera, on a tall stand to detect and record possible seizures/events with sufficient quality, the camera will be positioned in a way that will not disturb sleep and the equipment will not send out any disturbing light or radiation.

The patient recordings are stored on a Vodafone SIM card placed in our recording device. Algorithms in the device automatically detected abnormal events which are sent to cloud for further algorithmic analysis.


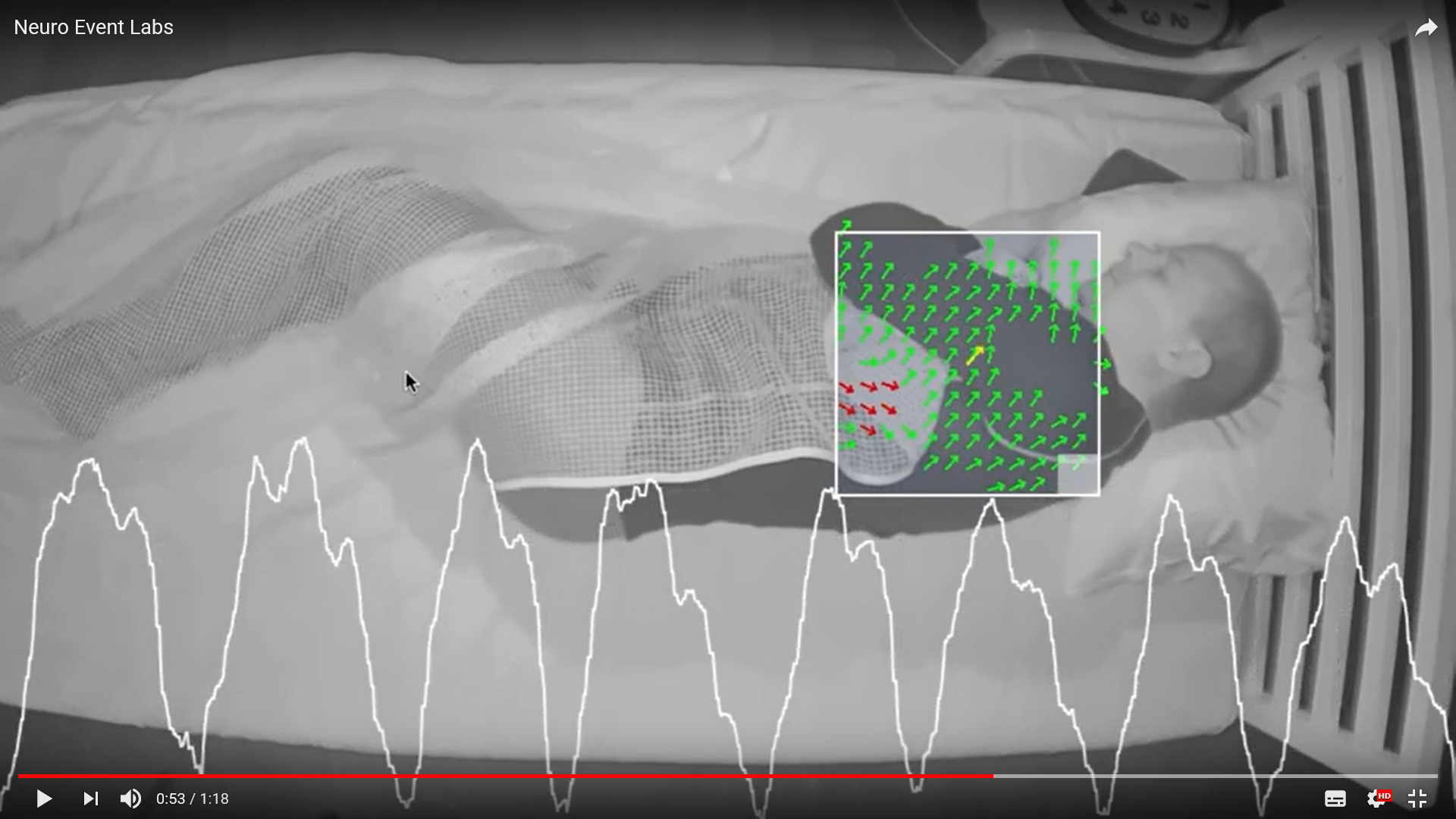


[**https://neuroeventlabs.com/for-physicians**](https://neuroeventlabs.com/for-physicians)
