# Supplementary figures and images for: Improving nocturnal event monitoring in people with intellectual disability in community using an artificial intelligence camera
Source: Epilepsy Behav Rep. 2023 Apr 23;22:100603. doi: 10.1016/j.ebr.2023.100603 (PMC10160340; doi:10.1016/j.ebr.2023.100603)

Supplementary information 3: Example of a Nelli report sent to professionals


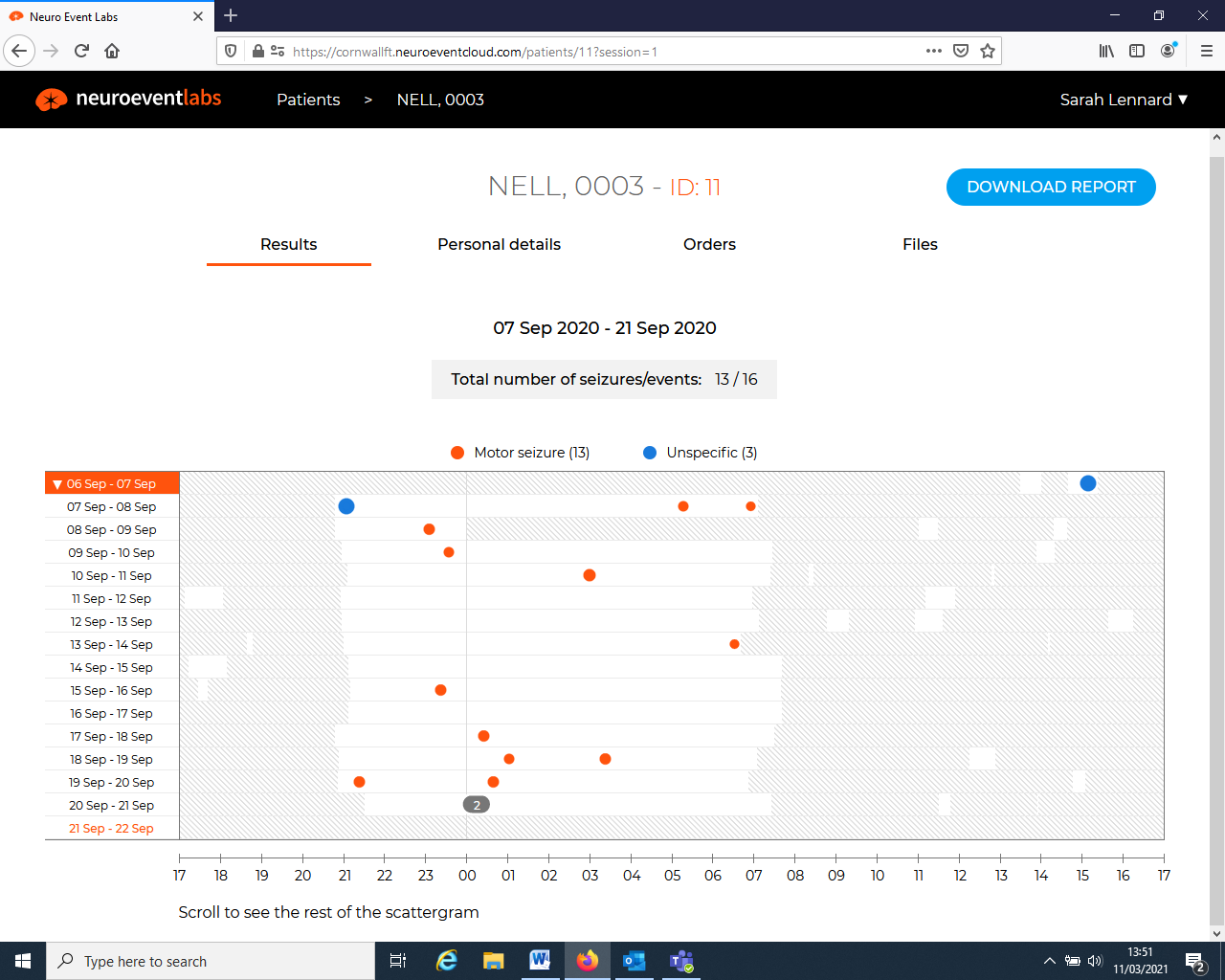


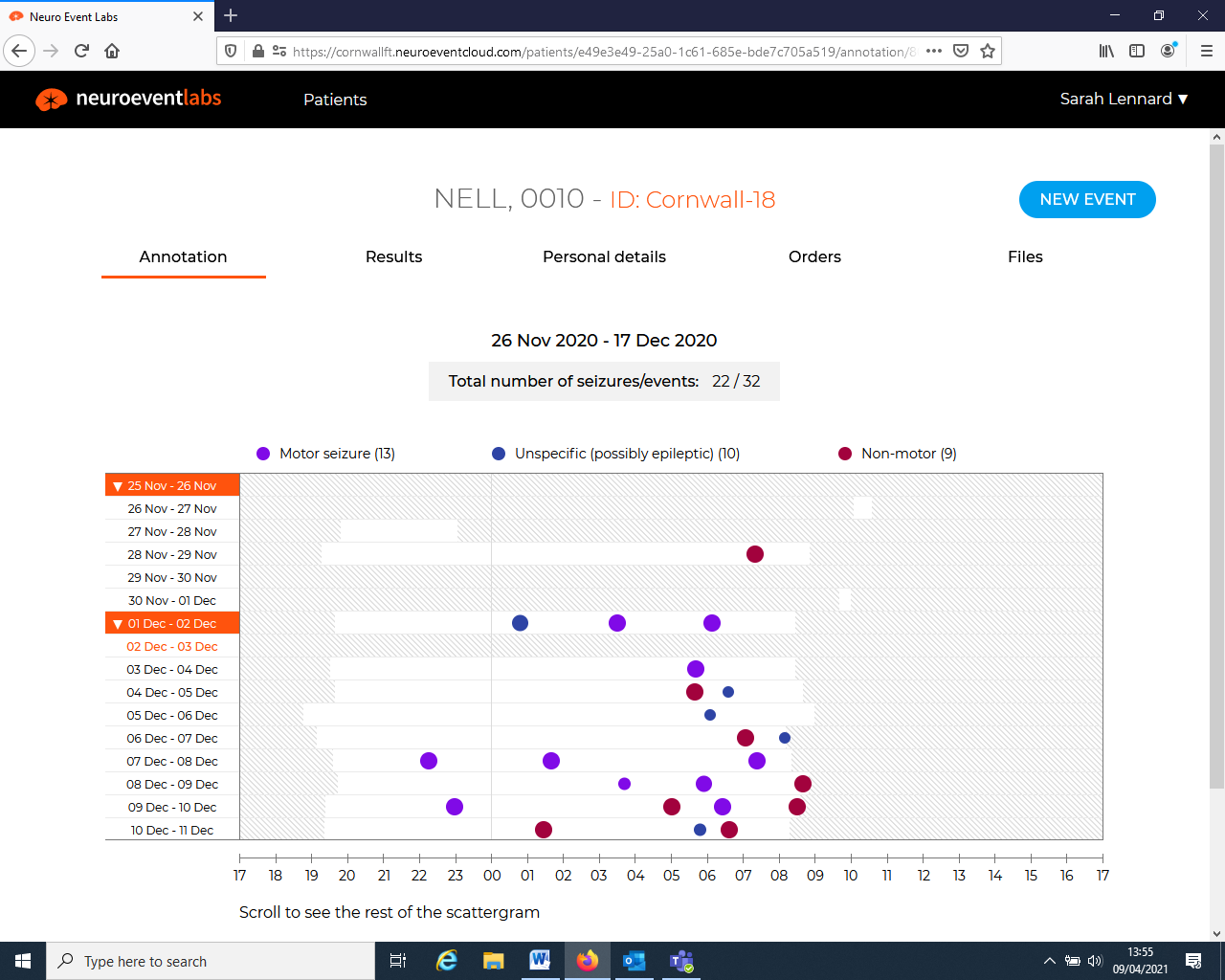


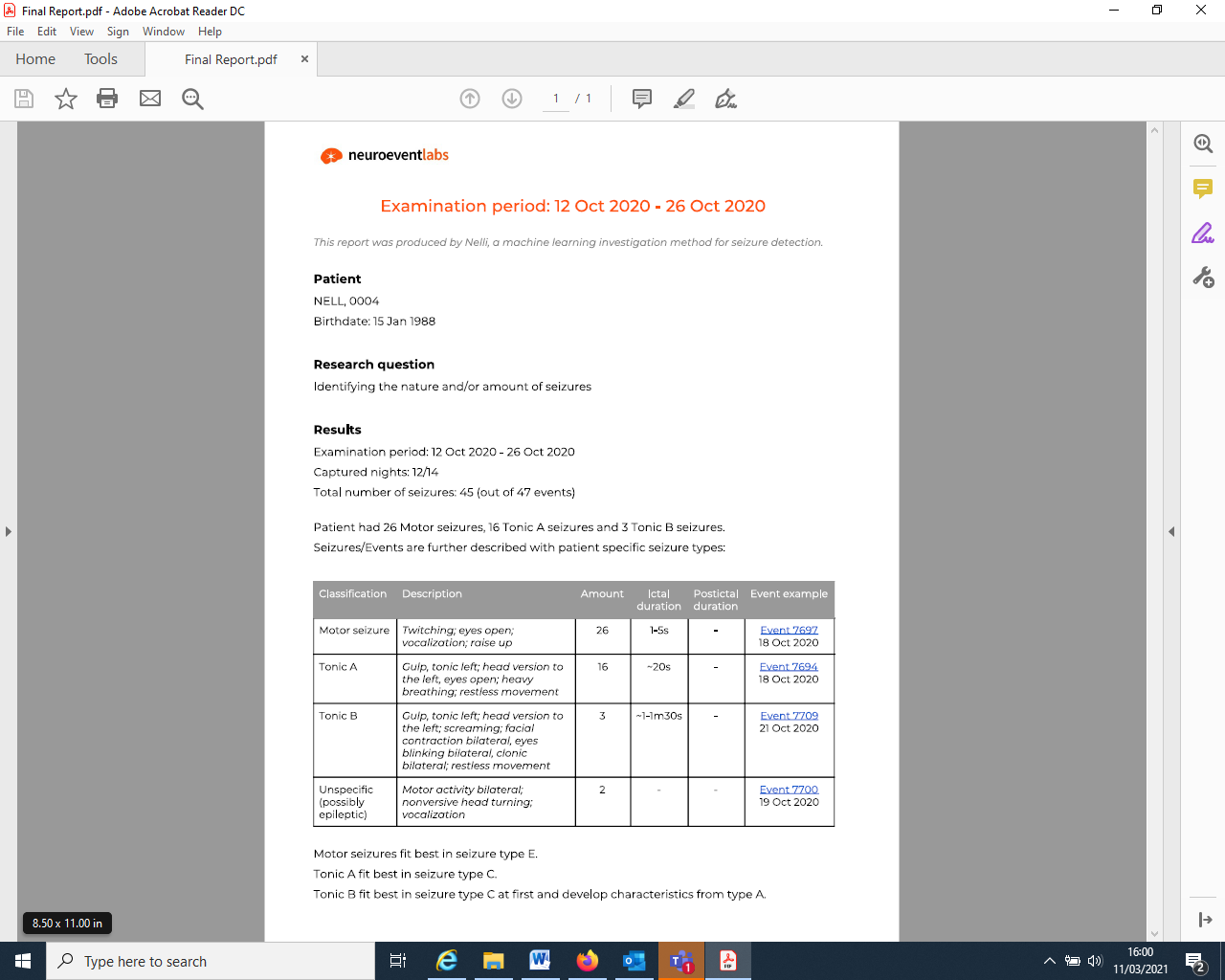


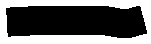

Supplement: Supplementary data 2 [file mmc2.docx]
