# Supplementary material for: Improving nocturnal event monitoring in people with intellectual disability in community using an artificial intelligence camera
Source: Epilepsy Behav Rep. 2023 Apr 23;22:100603. doi: 10.1016/j.ebr.2023.100603 (PMC10160340; doi:10.1016/j.ebr.2023.100603)
Supplement: Supplementary data 3 [file mmc3.docx]

Three

**Examples of verbatim feedback from questionnaire**

*Participant and Carer / family feedback*

*“The recording itself has shown others what was happening”*

*“Has highlighted that not all possible seizures* can/have been heard by sleep-in staff AND that some possible seizures cannot be determined by people who do not know the individual well”.*

*“It was really very shocking seeing the extent of the night-time possible seizures on one individual. Being able to clearly see how the possible seizures affected them, the recovery period and how they were trying to deal with what had happened in the dark on their own. The extent of the individual’s possible seizures was far worse than we had expected, and the recovery lasted for a lot longer than we realized. The individual was experiencing possible seizures regularly at night.”*

*“The results were shocking but informative. It should really help the support of this individual’s night as well as their day-to-day living. It has clearly shown everything that occurs at night... It has been interesting to see the results and how a person’s epilepsy can impact on their sleep pattern.”*

*Clinician Feedback*

“*Nelli allowed for each patient a comprehensive record of night events for a fortnight and helped us focus on areas of clinical interest and controversy. In several cases the quality of the images allowed us to confidently re-classify seizure events and amend management guidelines and re-evaluate clinical risks. The Nelli classification using the software was helpful in conjunction with clinical oversight from within our team”*

*“For the time of recording we were in several cases able to get improved information on all 3 of the above which was clinically important.”*

*“In one case made a diagnosis of epilepsy less likely (diagnosis was already in question) and is likely to affect treatment plan – may consider reducing rather than increasing antiepileptic drugs”*

*“Nelli helps focus clinician attention on episodes of interest and the classification is usually helpful, but not always the last word on classification. Clinical suspicion plus Nelli gives a more complete picture of the case.”*

* Where “seizures” have been mentioned by the respondent the authors have made it “possible seizures” to ensure we avoid confusion and clarify that it was not seizures but events which were possibly seizures.
