# Supplementary material for: Improving nocturnal event monitoring in people with intellectual disability in community using an artificial intelligence camera
Source: Epilepsy Behav Rep. 2023 Apr 23;22:100603. doi: 10.1016/j.ebr.2023.100603 (PMC10160340; doi:10.1016/j.ebr.2023.100603)
Supplement: Supplementary data 4 [file mmc4.docx]

Four

Adventures of Bob and

Nikolai the camera

I'm going to tell you a story about a camera called Nikolai. Nikolai lived in a beautiful home in Cornwall, with his friend Bob. Bob loved Nikolai, and they spent many years taking lots and lots of photos of all their adventures, family, and friends.

Nikolai had taken photos of Nana when she came to visit Bob, and of Uncle Sean and Uncle Steve, and all his friends who helped him at his home [say the names of the support workers on that day]; of walks in the Park, of the beaches and 'tunnel-water' trips in the car to see the animals at Longleat and going through the long dark tunnel for a day out in the city.

But, you see, Nikolai was getting old; his flash light had stopped working so no one wanted his photos anymore. Nikolai was very sad and asked Bob for some help...

“Bob...it's Nikolai the camera.”

“What's wrong Nikolai...?”

“I need some help.”

“What kind of help?”

“I need to go to the camera shop to get a new flash light so that I can see better when I take photos; that way people will want me to take photos again.”

“Come on then Nikolai, put your shoes on and your coat, it's a bit chilly today,

and let's go on a tunnel-water to the shops to look for your light.”

*Bob and Nikolai's adventure*

Nikolai the camera's very sad

He hasn't got a light, I bet he's mad

But Bob has a special plan for him

A 'tunnel-water' to the shops, to help his friend Nikolai, it'll all be fine when we

sing, when we sing this song...

Nikolai don't worry you'll be fine

Bob is going to help you find your light

I think he knows which way we where need to go

It's down the road, on the corner, could be left maybe right, all we need to do

is sing, is sing this song...

Nikolai, Bob found your light

It's just the right size and very bright

There's only one thing left for us to do

Is take a picture with your light, it'll be to everyone's delight, they'll be no

stopping you tonight, especially when we sing, we sing this song...

Nikolai the camera is very glad

He's helping Bob out, that can't be bad

He's taking lots of pictures all night long

To help the special doctors with their work, you'll be a star, on the night,

everything will be alright, especially when we sing, we sing this song...

So, Nikolai went home with Bob. They were both so happy after their tunnel water

shopping adventure. But Nikolai still needed people to take pictures.

Bob and Nikolai sat down together and made a plan. They had many ideas

then suddenly Bob said...

“I have a plan Nikolai.”

“Oh that's great! Tell me all about it Bob.”

“Well, my idea is that you take lots and lots of pictures of me Nikolai. You see

I've helped you, and now you can help me back.”

“Of course I'll help you. What do you want me to do?”

“Well, you see, my special doctors want to help me with my possible seizures but they need pictures of them. I'll let you stand in my room and with your special light you can take lots and lots of pictures of me.”

“What a great idea, and what a great plan you've made Bob! Of course I will help you.”

*It's great to make a plan*

It's great to make a plan

It's great to make a plan

Bob and Nikolai have made a plan

It's great to make a plan

It's great to make a plan

It's great to make a plan

Bob and Nikolai such fun they've had

Making a special plan

Lots and lots of photos

Of Bob I will take

To help the special doctors

It's going to be just great

Not one two three or even four

I'm going to help Bob

With many many more

It's great to make a plan

So Bob and Nikolai both helped each other. Nikolai the camera was happy

because he got a new light and was able to take great photos again. And,

Bob was happy because Nikolai was going to help the special doctors by

taking lots of photos to help them with their important work.

*Friends help each other*

When friends help each other

Plans come together

When friends help each other

They have fun together

Bob helped Nikolai

And Nikolai helped Bob

They both made a plan

Oh what fun they had

When friends help each other

They stick together

Because working together

Makes plans turn out better

Bob helped Nikolai

Nikolai helped Bob

They helped each other

They are superstars together

And so Nikolai was able to take lots of great photos again. And Bob helped the special doctors with their important work
